# President’s Address

**Published:** 1896-12

**Authors:** J. Y. Crawford

**Affiliations:** Nashville, Tenn.


### President's Address.

BY J. Y. CRAWFORD, M.D., D.D.S., NASHVILLE, TENN.

Abstract of a paper read before the American Dental Association, at Saratoga Springs, N. Y., August, 1896.

Notwithstanding the great financial depression and general agitation in our country, our profession has obtained considerable progress within the last twelve-months, it would be interesting and instructive, at this time, to relate and emphasize, but there are other matters which more directly affect the future welfare of our vocation and the good of the whole people that demand our attention. I desire to call your attention to the question of dental jurisprudence, or the legal enactments governing or regulating the practice of dental surgery in the United States.

I will say, first, that any enactment placed upon the statute-books of our common country, or any of the independent States of our common country, should rest upon the immutable principle of equal and exact justice to all, with exclusive privileges to none.

Second. All laws are intended to control the conduct of an individual, or individuals in regard to any definite and universally uniform question in all the States, should be absolutely the same in all particulars.

Third. All laws should be so plain and simple in their construction that the humblest citizen could comprehend them, and, if necessary, administer the same in case he is called upon to do so by the suffrages of the people.

Fourth. The penal feature should be so well marked, and so imperative in demand that none would dare to disregard, only at their peril.

Fifth. Each State should have the same enactments controlling the matter of dental education, making the requirements for graduation uniform in every particular, thus holding dental colleges up to a higher and better standard.

Sixth. The first registration of an individual to practice should be made within twelve months after receiving a diploma from a regular dental institution of learning. Subsequent registrations in any other State should depend upon the presentation of a certificate from the State Board of Dental Examiners, that the individual held a diploma from a reputable dental college, and that he had engaged only in the reputable practice of dental surgery while remaining a citizen of the State in which he first registered.

In order that this be accomplished Dr. Crawford suggested that the American Dental Association, the Faculties Association and the Association of Dental Examiners, each appoint one member from each State to constitute a committee.

Further, he said: It is certainly evident that unless something be done in the direction of strengthening the moral force of the laws governing the practice in many of the States, the effect of such enactments now upon our statute books will become nil, so far as the desired results are concerned.

We should encourage the uniformity of such laws in all of our States which have the same definite and legitimate object in view. The common patriotism of any learned profession should be willing to do, at least, this much in the advancement and development of the proper fraternal feeling in all the States, thus doing something in the line of obliterating all sectional lines and objectionable sectionalism.

There are many citizens, good and reputable, now engaged in the practice of dentistry in some States who are deprived of citizenship in other States by virtue of the improper construction and requirements of some of the laws in States of this Union, thus virtually disfranchising them to a certain extent. I would insist, epecially, that the laws governing dental legislation be so constructed that no college would be regarded as reputable and entitled to public confidence, or be authorized to issue a legal diploma which habitually and knowingly admitted students, or other persons, who have been illegally engaged in the practice of dental surgery in any State.

Dr. Crawford further spoke of the National Dental Museum, and the consolidation of the Southern and American Dental Societies, suggesting that, if this be accomplished, the meetings be held in three grand divisions of our country: the north-east to have one meeting, the north-west the next, the south the next, and so on, alternating from year to year, that the whole profession throughout the entire country may have its full share and benefit resulting from our national organization.

The committee on this address reported that while they realized the importance and needs of such legislation they doubted the practicability of securing absolutely uniform legislation in any large number of States within the near future, and were also in doubt as to the expedincy of appointing, at the present time, a committee to be charged with such an undertaking.

